# Social Cognition Individualized Activities Lab for Social Cognition Training and Narrative Enhancement in Patients With Schizophrenia: A Randomized Controlled Study to Assess Efficacy and Generalization to Real-Life Functioning (Prot. n°: NCT05130853)

**DOI:** 10.3389/fpsyt.2022.833550

**Published:** 2022-04-04

**Authors:** Davide Palumbo, Edoardo Caporusso, Giuseppe Piegari, Claudio Mencacci, Sara Torriero, Luigi Giuliani, Michele Fabrazzo, Dario Pinto, Silvana Galderisi

**Affiliations:** ^1^Department of Psychiatry, University of Campania “Luigi Vanvitelli,” Naples, Italy; ^2^Department of Psychiatry and Addiction, ASST Fatebenefratelli-Sacco, Milan, Italy

**Keywords:** social cognition training, narrative enhancement, metacognition, real-life functioning, schizophrenia

## Abstract

Subjects affected by schizophrenia present significant deficits in various aspects of social cognition, such as emotion processing, social perception and theory of mind (ToM). These deficits have a greater impact than symptoms on occupational and social functioning. Therefore, social cognition represents an important therapeutic target in people with schizophrenia. Recent meta-analyses showed that social cognition training (SCT) is effective in improving social cognition in subjects with schizophrenia; however, real-life functioning is not always ameliorated. Integration of SCT with an intervention targeting metacognitive abilities might improve the integration of social cognitive skills to daily life functioning. Our research group has implemented a new individualized rehabilitation program: the Social Cognition Individualized Activities Lab, SoCIAL, which integrates SCT with a module for narrative enhancement, an intervention targeting metacognitive abilities. The present multi-center randomized controlled study will compare the efficacy of SoCIAL and treatment as usual (TAU) in subjects diagnosed with a schizophrenia-spectrum disorder. The primary outcome will be the improvement of social cognition and real-life functioning; while the secondary outcome will be the improvement of symptoms, functional capacity and neurocognition. The results of this study will add empirical evidence to the benefits and feasibility of SCT and narrative enhancement in people with schizophrenia-spectrum disorders.

## Introduction

In subjects with schizophrenia an impairment in different cognitive domains has been well established (1–9). Cognitive impairment has been associated with poor social functioning and poor quality of life (10–14). Evidence has been reported suggesting that the impact of cognitive impairment in real-life functioning is even greater than the one exerted by positive and negative symptoms (15–19). Furthermore, this impairment can be detected throughout different phases of schizophrenia, such as the premorbid, prodromal and remission stages of the illness (20–25). From a neurobiological perspective, this impairment might be explained as the cumulative effect of abnormalities in neuronal maturation processes, neurodevelopment and neuroplasticity that could lead to defective cortico-cerebellar-thalamic-cortical circuits (26–29).

Social Cognition (SC) is a complex construct that underlies fundamental abilities for social interactions, such as the capacity to perceive, interpret and generate answers based on other people’s intentions, emotions, and behaviors (4, 30–32). The ability to form integrated ideas about oneself, others, and the world based on SC, i.e., metacognition, is essential for the integration of the social cognitive skills to daily life functioning. Both SC and metacognition concur to construct a coherent narrative on internal experiences and behavior in different interpersonal situations (30, 31, 33).

Among all the cognitive domains, SC was highlighted not only for its direct impact on real-life functioning, but also for its role in mediating the effects of other cognitive domains on functioning (15, 17, 34, 35).

Studies regarding SC in schizophrenia identified 4 sub-domains impaired in affected subjects: emotion recognition, social role perception, theory of mind (ToM) and attributional style (30, 31, 36).

Despite the key role that cognitive impairment plays in schizophrenia, pharmacological treatments seem to have a marginal impact (2, 37, 38); consequently, a number of psychological treatments has been developed in order to get a significant improvement of several cognitive domains, quality of life and real-life functioning (39–46). Specifically, the cognitive remediation programs were developed mainly to target neurocognitive functions (such as attention, memory, learning) rather than SC, which only in the last decade became the target of specific training programs with promising results (30, 47–49).

Different approaches have been used to develop cognitive remediation programs for SC: some of them, defined as “integrated,” aimed to train both SC and neurocognitive domains; other programs, defined as “targeted,” are designed to improve only specific SC domains (50–53). A meta-analysis of SC programs showed that these interventions produce significant improvements in emotion recognition and ToM, as well as in real life functioning and in negative symptoms (49). According to the results of a recent meta-analysis, the inclusion of a training for neurocognition does not add any advantage (53). Furthermore, the meta-analysis also showed that generalization to real-life functioning of the improvement is not always obtained.

None of the social cognition training (SCT) programs included a focus on metacognition, specifically oriented to mediate and facilitate the transfer of therapeutic gains to real-life tasks.

The Department of Psychiatry of the University of Campania “Luigi Vanvitelli” developed an integrated group intervention, the Social Cognition Individualized Activities Lab (SoCIAL), with the purpose to improve SC domains (recognition of emotions and ToM). In the pivotal study, SoCIAL was compared to a largely validated remediation program for social abilities, the Social Skills and Neurocognitive Individualized Training (54–56). Results showed that participants who received the SoCIAL program improved their SC abilities, together with a significant reduction of the avolition domain in negative symptomatology, compared to the control group (56).

Conversely, no difference was found among the two treatment groups when looking at other clinical variables, such as positive and disorganized symptoms, neurocognitive functions and real-life functioning. In particular, both groups did not show an improvement in social functioning, probably due to a ceiling effect, i.e., the high level of functioning that these patients already had before the treatment started (56).

The main limitations found for this program concerned two aspects: (1) the group setting and (2) the role-play module. The group setting was not accepted by all subjects and participants’ recruitment was challenging as some subjects refused or were unable to attend the group sessions. The role-play, a very useful technique for the development of social abilities, is less effective in training SC, as subjects with greater SC deficits in the group may experience frustration during the role-play if other subjects present milder levels of SC deficits. The individual format of the SoCIAL training was found to be accepted by all subjects and allowed a better personalization of the SC training.

Additionally, to favor the transfer of therapeutic gain to real-life functioning we added an intervention on metacognition, focused on understanding the role of emotions and intentional states in real-life situations, to develop a coherent narrative about self and others. The literature has recently identified an interesting technique that could help individuals affected by schizophrenia train their narrative ability, i.e., *narrative enhancement* (57–63). Several studies have shown a correlation between narrative abilities and metacognition, i.e., the ability to integrate newly acquired cognitive skills to daily life functioning (64–66). Narrative deficits play a substantial role in determining the poor quality of life for people with schizophrenia and are associated with negative and depressive symptoms (65, 67). Some studies (57, 58) used the *narrative enhancement* technique in a rehabilitation program defined as “integrated,” since it trained both neurocognitive and narrative abilities. The results are encouraging, considering that this technique seems to considerably decrease internalized stigma and to improve quality of life of participants, even when compared to other psychological and rehabilitative programs (58).

The present protocol aims to evaluate the efficacy of the integrated SoCIAL program in a large sample of individuals with schizophrenia-spectrum disorders. The program will be administered individually and will include the *narrative enhancement* module, specifically developed to target metacognitive abilities to recognize and integrate mental states, such as emotions, desires, attitudes and goals in real-life situations. The efficacy of the program will be evaluated by comparing the participants randomized to SoCIAL with those randomized to treatment as usual (TAU). The use of the TAU control was deemed appropriate as the study is an early phase trial in which an active control condition including different treatment components may reduce power and increase type II error (68).

The study aims to compare the efficacy of the SoCIAL program vs. TAU in subjects with schizophrenia or schizoaffective disorder (based on DSM-5 criteria) with illness durations < 10 years. The primary outcome is the improvement of social cognition and real-life functioning; while the secondary outcome is the improvement of negative symptoms, functional capacity and neurocognition.

## Materials and Methods

### Subjects

The study will be carried out at the Department of Psychiatry of the University of Campania “Luigi Vanvitelli” in Naples, and the Department of Mental Health and Addiction Services, ASST Fatebenefratelli Sacco in Milan. Eighty consecutive outpatients with a diagnosis of schizophrenia/schizoaffective disorder according to DSM-5 criteria, confirmed by the Structured Clinical Interview for DSM-5-Patient version (SCID-I-P) will be enrolled, 40 in each center.

Inclusion criteria are: (1) age between 18 and 50 years; (2) clinical stability defined as no major pharmacological treatment modifications in the last 3 months; (3) illness duration < 10 years; (4) at least 5 years of school education. Exclusion criteria are: (1) medical conditions that cause disabilities; (2) a history of alcohol and/or substance abuse.

Subjects will be considered “drop out” when: (1) they do not participate in the study sessions for 3 consecutive weeks, or withdraw the consent to continue the study; (2) they do not participate in the study sessions for 3 consecutive weeks, re-join the program and then accumulate 2 more weeks of absence from the study (in total 5-week interruption of the program, even when non-consecutive); (3) worsening in psychopathological condition requiring a substantial modification of the pharmacological therapy and/or a hospitalization.

Subjects will be randomly assigned to the SoCIAL or the TAU group. Overall, 40 patients (20 for each center) will be assigned to SoCIAL and 40 (20 for each center) to the TAU. Subjects will be evaluated before starting the program and at the end of the intervention.

The study will be approved by the Ethics Committees of the two centers. All enrolled subjects will sign an informed consent form, approved by the Ethics Committees.

### Randomization

Following eligibility and baseline assessment participants will be randomly assigned to either the intervention or control (TAU) group using an *ad hoc* Excel spreadsheet (69). We will stratify randomization by age, gender and years of education.

### Assessment Instruments

#### Psychopathology

The Positive and Negative Syndrome Scale (PANSS, administration time = 30’) will be used for the evaluation of positive, negative and disorganized symptoms (70). The PANSS is a semi structured interview composed of 30 items divided in three subscales, namely positive symptoms, negative symptoms, and general psychopathology. Each item is accompanied by a specific definition and by detailed anchoring criteria for each rating point, ranging from “absent” (1) to “severe” (7) (71, 72).

The negative symptom subscale includes items which assess cognitive functions and disorganization, therefore only the core negative symptoms will be included in the summary score according to the recently published European Psychiatric Association (EPA) guidance paper on the assessment of negative symptoms (71).

#### Neurocognitive Functions

Neurocognitive functions will be assessed using the Measurement and Treatment Research to Improve Cognition in Schizophrenia (MATRICS) Consensus Cognitive Battery (MCCB) (73–75). This test includes items designed to evaluate seven cognitive domains: (a) processing speed; (b) attention and vigilance; (c) working memory; (d) verbal learning and memory; (e) visual learning and memory; (f) social cognition; (g) reasoning and problem solving (administration time = 80’). The MCCB includes 10 neuropsychological tests (Category Fluency—Animal Naming; Brief Assessment of Cognition in Schizophrenia Symbol Coding; Trail Making Test—Part A; Continuous Performance Test—Identical Pairs; Wechsler Memory Scale Spatial Span; Letter-Number Span; Hopkins Verbal Learning Test—Revised; Brief Visuospatial Memory Test—Revised; Neuropsychological Assessment Battery—Mazes; Mayer-Salovey-Caruso Emotional Intelligence Test) (73, 74). General cognitive abilities will be evaluated with the short form of the Wechsler Adult Intelligence Scale-Revised (WAIS-R). The short form of the WAIS-R uses four subtests (arithmetic, block design, picture completion, and information) to estimate verbal and performance IQ that are highly correlated with the full WAIS assessments (76).

#### Social Cognition

To better assess social cognition (in addition to the social cognition test included in the MCCB) participants will be administered the Facial Emotional Identification Test (FEIT, administration time = 15’), as well as The Awareness of Social Inference Test (TASIT, evaluation time = 45’), an emotion processing and ToM measure. The FEIT involves black-and-white photographs of 19 different individuals’ faces (nine females/10 males) each depicting one of six different emotions (happiness, sadness, anger, surprise, fear, shame), shown one at a time for 15 seconds (77). After each stimulus, the participant should select which of the six emotions better describe the picture shown (78). The Awareness of Social Inference Test (TASIT) is a measure of basic emotion perception (section “Introduction”) and complex social cognition (ToM in simple and complex interpersonal situations involving sarcasm and deceptions). This test evaluates social cognition through videotaped vignettes designed to reflect real life interactions in different contexts, including free time and work situations (79, 80).

#### Functional Capacity and Real-Life Functioning

Functional ability will be assessed by the brief version of UCSD Performance-based Skills Assessment (UPSA-Brief, administration time = 15’), an instrument that measures participants’ capacity to perform tasks similar to those encountered in daily life (81–83). The UPSA-B consists of two of the five subscales from the full UPSA: (1) Financial skills and (2) Communication skills. For the assessment of financial skills, participants are given tasks matching everyday life situations (for example, they are asked to count change or are asked to pay bills). Assessment of communication skills involves tasks in which participants use a disconnected landline telephone to simulate phone calls (e.g., to the doctor’s office) to communicate necessary information. The final score (calculated by summing the two subscale scores) ranges from 0 to 100, with higher scores indicating better functional capacity (81). The Specific Level of Functioning Scale (SLOF, administration time = 20’) will be used to evaluate the subject’s functioning (10). This scale consists of 43 items and relies on data reported by a caregiver or a caseworker of the examined subject (selected on the basis of their familiarity with the subject) and based on the direct observation of subject’s behavior and functioning in several domains: (1) physical functioning, (2) personal care skills, (3) interpersonal relationships, (4) social acceptability, (5) activities of everyday life and (6) work skills (10). Quality of life will be measured through the Quality of Life Scale (QoLS, administration time = 15’), a semi-structured interview, composed by 21 items, designed to assess four different areas of psychosocial adjustment, including Interpersonal Relations, Instrumental Role (e.g., work, school, homemaker), Intrapsychic Foundations (e.g., motivation, sense of purpose), and Common Objects and Activities (e.g., owning a watch, use of public transportation) (84).

### Individualized Cognitive Remediation Program (SOcial Cognition Individualized Activities Lab, SoCIAL)

The SoCIAL program will be administered once a week, for a total of 10 weeks. Each session consists of two different modules: (1) a training program that helps patients recognize emotions and important social signals (such as facial expressions and prosody) and develop strategy focused on the ToM; (2) the *narrative enhancement* module to train metacognitive abilities. Time of administration for both modules is 30 min; the operator, however, can choose to focus the session on one module rather than the other, based on the subject’s specific needs.

Hereafter the description of the two modules.

#### Social Cognition Training

**FIGURE 1:**
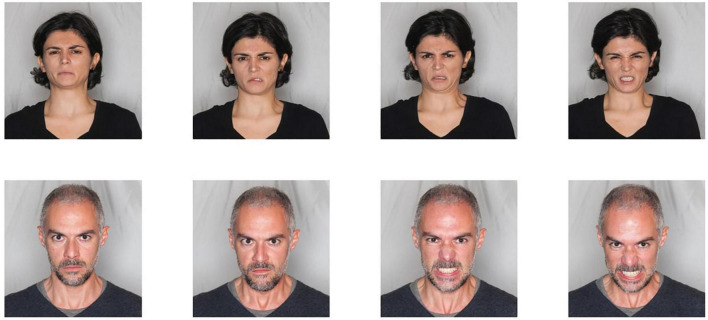
Facial emotion recognition material. From top left to top right: increasing intensity of disgust in a woman face; from bottom left to bottom right: increasing intensity of rage in a man face.

**FIGURE 2:**
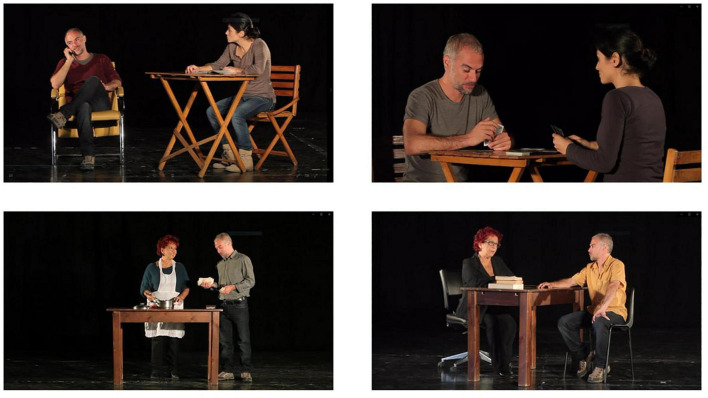
Frames of social interaction’s videos.

The Emotions recognition training will train the subject’s ability in discriminating between different emotional states; it consists of two sessions for each basic emotion (10 sessions total): fear, anger, surprise, sadness and joy. Educational materials (photos and videos) are used to illustrate prototypical elements of each emotion (facial micro expressions, prosody, gestures etc.) (56). Patients are trained to identify signs of facial emotions using both static faces (photographs) (Figure 1) and dynamic situations (videos) (Figure 2). Lastly, patients will be presented faces with ambiguous emotion expressions and they will be trained in emotions recognition using video footage of daily activities characterized by ambiguous emotional activations.

**FIGURE 3:**
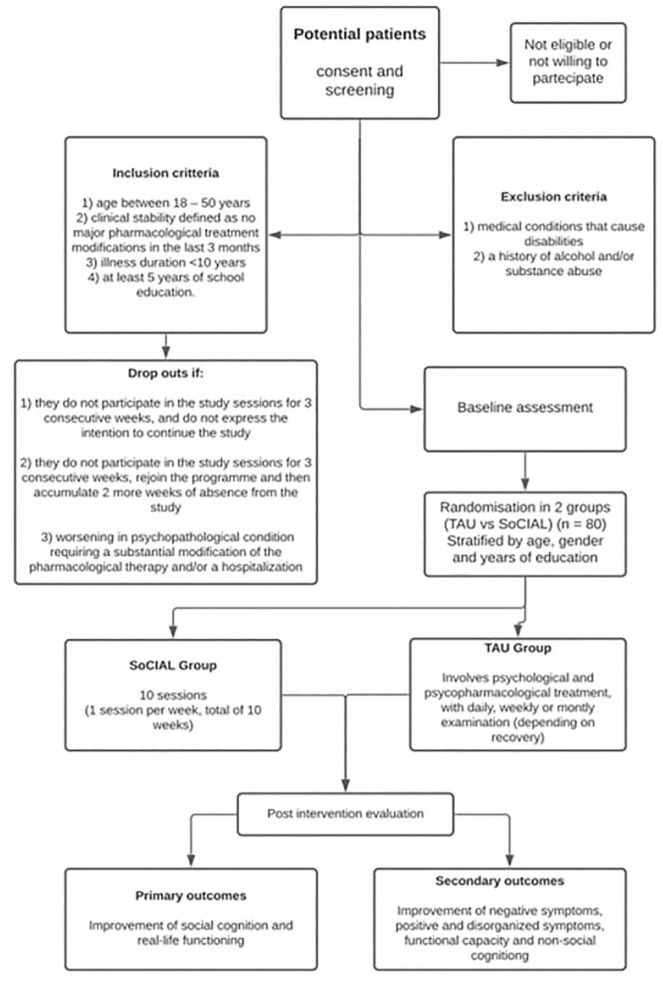
Study flowchart: TAU: treatment as usual.

The ToM training will help subjects discriminate between emotional expressions in social contexts and understand other people’s mental state (the ability to figure out what other people think and feel in social situations in different contexts, such as work or interpersonal). The ToM training consists of 10 sessions. In each session subjects will watch videos (Figure 3) that display actors expressing one of the following: sarcasm, disappointment, hostility, misunderstanding, deceit and sincerity. The subject will be trained to recognize the feelings, intentions and meaning of the actors’ behaviors in the videos, based on their facial expression, tone of voice and gestures (56). This module uses videos representing social interactions that are increasingly difficult to understand. These tasks will require higher abilities compared to the emotion recognition training, considering that subjects have to integrate verbal and non-verbal emotional data as well as comprehend other people’s mental states in different social situations (e.g., use of the sarcasm in interpersonal work situations).

Further information about how the training material was developed can be found in Palumbo et al. (56).

#### Narrative Enhancement Module

The narrative enhancement training is based on the assumption that subjects with schizophrenia show a wide range of impairments in self-awareness, self-reflection, and narrative abilities (64–66, 85–88). The narrative enhancement training in the SoCIAL program consists in story-telling exercises focused on improving the subject’s capacity to comprehend his/her emotional experience and integrate emotions and behavior in real-life situations. The operator uses a semi-structured interview procedure. In the first phase, he asks the patient to share a personal story that makes him/her suffer (e.g., Do you want to tell me a personal story that makes you suffer?). After collecting the story, in a second phase, the therapist clarifies with the patient the specific emotions that the patient feels/has felt in relation to the story (e.g., While you were living this experience, what emotions did you feel? Now how do you feel reliving this experience?). In the third and final phase, the therapist and patient question the emotions felt and whether these emotions can influence the patient’s actions even in the immediate present or in the near future (e.g., Is it possible that feeling inadequate at that party led you to avoid other similar situations?).

### Treatment as Usual

Subjects that will be randomized in this group will receive their TAU for the whole length of the study. TAU includes all the ongoing psychiatric therapies (pharmacological, psychological, occupational, etc.) that subjects may have begun before study’s enrollment.

The number of therapy hours will be recorded and will represent a covariate in group comparisons.

### Length of the Study

The study will have a total length of 12 months, divided in the following phases.

Phase 1. Operators will be trained in evaluating neurocognitive functions, social cognition, functional capacity and real-life functioning. Phase 2. Different operators will be trained in SoCIAL program’s practice. Phase 3. Recruitment of patients and randomization (in both centers). Phase 4. Assessment of neurocognitive functions, social cognition, functional capacity and real life functioning for enrolled patients. Phase 5. Implementation of the interventions. Phase 6. Assessment of neurocognitive functions, social cognition, functional capacity and real life functioning in subjects at the end of the program for both groups (SoCIAL program vs. TAU). Phase 7. Final evaluation and data analysis.

The study’s timetable is shown in Table 1 and the flowchart in Figure 3.

**TABLE 1:** Time schedule of the study.

	Enrollment	Pre-therapy assessment	Allocation	Intervention	Post-therapy assessment
**Enrollment**					
SCID 5	X				
WAIS-R	X				
Informed consent	X				
Randomization					
Allocation			X		
**Intervention**					
SoCIAL				X	
TAU group					
**Assessments**					
PANSS		X			X
MCCB		X			X
FEIT		X			X
TASIT		X			X
UPSA-B		X			X
QoLS		X			X

*SCID 5, Structured Clinical Interview for DSM-5 - Patient version; WAIS-R, Wechsler Adult Intelligence Scale revised; TAU, Treatment as usual; PANSS, positive and negative syndrome scale; MCCB, Matrics consensus cognitive battery; FEIT, facial emotion identification test; TASIT, The Awareness of Social Inference Test; UPSA-B, UCSD Performance-based Skills Assessment; QoLS, Quality of Life Scale.*

### Data Analysis

An assessment of the statistical power of this study was performed using an effect size analysis of the studies included in a meta-analysis (49). The results are very satisfactory: with a statistical power ≥ 0.8 and a significance level of < 0.05, our study needs a total sample of 68 subjects largely below the 80 subjects that will be recruited.

Neuropsychological, psychopathological and cognitive functioning evaluations will be conducted pre and post interventions. Statistical analysis will be conducted by ANOVA for repeated measures, focusing on the role played by different types of interventions in each group regarding the aforementioned domains.

## Conclusion

SoCIAL is an individualized rehabilitation intervention, specifically designed to improve SC and metacognitive abilities in patients with schizophrenia or schizoaffective disorder. Therefore, this program is meant to train only SC and metacognition, leaving out the other cognition domains. In the recent literature it is still debated whether a combined intervention (i.e., that trains neurocognition using a cognitive remediation training, CRT, along with SC) (89) is better than SC training, as the combined intervention leads to greater improvements in different cognitive domains and in social cognition than CRT alone, a recent meta-analysis indicates that CRT does not enhance the effects of SC training (53). In our opinion, these results (53, 89) indicate unambiguously that the active ingredient to improve social cognition is SCT. Our program consists of two modules: 1) a module that trains emotion recognition and ToM and 2) a module that trains the narrative abilities, focused on metacognition. The SoCIAL program is the first intervention that trains both SC and narrative abilities in subjects with schizophrenia or schizoaffective disorder. To our knowledge, very few studies investigated the effect of narrative enhancement in this population of patients (64–66). In particular, this program, compared to others, shows some peculiarities in narrative enhancement. Firstly, it will be administered individually (not in a group setting); the individual setting allows the possibility of exploring individual experiences and possible difficulties in understanding one’s own emotions in relation to that experience. Secondly, in different narrative enhancement programs patients are asked to write and share stories regarding their illness. The goal of these interventions is to reduce the self-stigma associated with the disease. In SoCIAL, instead, patients are allowed to pick up a topic of their choice for the narrative enhancement training, including those related to internalized stigma. The goal is to train narrative abilities focusing on developing metacognitive skills in integrating internal experience and mental states with behavior in a coherent narrative. The module is added to transfer therapeutic gains of the SCT to real-life functioning. The SCT and narrative enhancement are expected to potentiate each other in developing emotion recognition and ToM skills and to develop narrative abilities.

Upon completion of data collection, it is expected that participants who receive the SoCIAL intervention vs. the TAU group will improve social cognition, social functioning and quality of life. Lastly, this study aims to evaluate if this intervention could lead to improvements in positive, negative and disorganized symptoms of schizophrenia, together with functional capacity and cognition.

The results of this study will add empirical evidence to the benefits and feasibility of SCT and narrative enhancement in people with schizophrenia-spectrum disorders.

## Limitations

The design of this study should be examined considering some limitations. First, a follow-up evaluation should be added in future studies to address persistence over time of the gain in social cognition and real-life functioning. Second, in our study the efficacy of the SoCIAL intervention is evaluated only vs. TAU. As other trials on SCT efficacy had used an active control (90), the inclusion of a TAU control might be seen as a limitation of the study. However, as we try to prove the efficacy of an innovative intervention integrating SCT with narrative enhancement to further improve the gain in real-life functioning, only a TAU control was included to avoid type II error. If our study will demonstrate an effect on social cognition and real-life functioning, the next step will be a phase 2 trial in a large group of subjects randomized to either SoCIAL or an active control group in which only the SCT component will be provided together with traditional homework tasks, controlling for the number of sessions, individualization of therapy, delivery of therapy fidelity procedures, as well as aspecific factors such as motivation of patients and therapists. This could clarify whether the narrative enhancement module is essential for the transfer of therapeutic gain to real-life functioning.

Lastly, our study used FEIT and TASIT as measures of social cognition, although there is a lack of consensus regarding the use of FEIT for the assessment of emotion recognition. However, the results of the Italian Network for Research on Psychoses studies clearly showed the validity of the test in catching the deficits in social cognition of subjects with schizophrenia (6, 10, 15, 34, 91). We believe that its output, corroborated by the TASIT section of emotion recognitions, shall be considered solid.

Another aspect that could be seen as a limitation of our protocol is the lack of inclusion of a social competence measure which could be improved even if no changes in real-world outcomes are detected. However, no study in our knowledge demonstrated an improvement of social competence by SCT and our own data demonstrated that social cognition impact on real-life functioning is mediated in part by functional capacity, as measured using the UPSA-B, which is included in our protocol. Furthermore, the network analysis by our research group showed that social cognition had no link to an included measure of social competence (6, 92).

## Ethics Statement

The protocol has been approved by the Ethics Committee of the Università degli Studi della Campania “Luigi Vanvitelli” – A.O.U. “Luigi Vanvitelli,” A.O.R.N. “Ospedale dei Colli” and by the Ethics Committee of ASST Fatebenefratelli-Sacco of Milan. Written informed consent was obtained from the individuals for the publication of any images or data included in this article.

## Author Contributions

SG, DPa, and GP initiated the project idea. DPa planned the experimental procedures. DPa and EC drafted the manuscript. All authors contributed to critically revising the content and approved the final manuscript.

## Conflict of Interest

The authors declare that the research was conducted in the absence of any commercial or financial relationships that could be construed as a potential conflict of interest.

## Publisher’s Note

All claims expressed in this article are solely those of the authors and do not necessarily represent those of their affiliated organizations, or those of the publisher, the editors and the reviewers. Any product that may be evaluated in this article, or claim that may be made by its manufacturer, is not guaranteed or endorsed by the publisher.
